# m6A-dependent up-regulation of DRG1 by METTL3 and ELAVL1 promotes growth, migration, and colony formation in osteosarcoma

**DOI:** 10.1042/BSR20200282

**Published:** 2020-04-21

**Authors:** Zhian Ling, Liangjun Chen, Jinmin Zhao

**Affiliations:** 1 Department of Orthopedics, The First Affiliated Hospital of Guangxi Medical University, Shuangyong Road, NO.22, Nanning, Guangxi 530021, China; 2 Department of Orthopedics, The Second Affiliated Hospital of Guangxi Medical University, University East Road, NO.166, Nanning, Guangxi 530007, China

**Keywords:** 143B, MG63, reader protein, RIP-qPCR, western blot, writer protein

## Abstract

Osteosarcoma (OS) is a malignant tumor commonly observed in children and adolescents. Developmentally regulated GTP-binding protein (DRG) 1 plays an important role in embryonic development; aberrantly expressed DRG1 has been associated with pathological processes in cancer. The present study aimed to explore the role of DRG1 in OS and the mechanisms underlying DRG1 overexpression in OS. Clinical studies were performed to evaluate Drg1 expression in OS tissues and to identify a correlation between Drg1 expression and the clinicopathological features in patients with OS. Drg1 was knocked down in OS cells to determine its effects on cell viability, cell cycle distribution, apoptosis, migration, invasion, and colony formation rate. METTL3 and ELAVL1 were also silenced to determine their effects on the levels of N6-methyladenosine (m6A), RNA stability, and Drg1 expression. Higher levels of Drg1 mRNA and protein were observed in OS tissues than those in paracancerous tissues. High expression of DRG1 was associated with large tumor size and advanced clinical stages in OS. Silencing of Drg1 resulted in decreased viability and inhibition of the migration and colony formation abilities of OS cells; it also resulted in cell cycle arrest in the G_2_/M stage and induced apoptosis. Knockdown of METTL3 led to decreased m6A and Drg1 mRNA levels. ELAVL1 knockdown impaired the stability of DRG1 mRNA, thereby reducing both the mRNA and protein levels of DRG1. In all, DRG1 exerted tumorigenic effects in OS, and the up-regulation of DRG1 in OS was induced by METTL3 and ELAVL1 in an m6A-dependent manner.

## Introduction

Osteosarcoma (OS) is a malignant bone tumor, commonly arising in the metaphyseal ends of long bones in children and adolescents. The incidence of OS is globally increasing year by year, accounting for approximately 35% of the primary malignant bone tumors [1,2]. However, the rate of survival for patients with OS is still poor, especially for patients with metastatic OS [1,2]. Lack of complete elucidation of the pathogenesis of OS has hindered the development of effective OS treatment.

Developmentally regulated GTP-binding protein (DRG) 1—also termed as NEDD3—belongs to the GTP-binding protein superfamily. It is a highly conserved gene that is expressed in almost all tissues. DRG1 plays an important role during embryonic development [3–5]. Although DRG1 is down-regulated after birth, high expression of DRG1 is observed in rapidly growing tissues and organs [6]. Studies have found that the function of DRG1 is regulated by various signals, including hypoxia, DNA damage, homocysteine, androgens, and N-Myc [7–9].

A growing number of studies have shown that abnormal expression of DRG1 is associated with occurrence and development of cancer. In lung adenocarcinoma, overexpressed DRG1 increased cell proliferation by regulating cell cycle proteins and enhanced resistance against paclitaxel (Taxol) [10]. shRNA-mediated knockdown of DRG1 inhibited melanoma cell proliferation and consequent colony formation [11]. These data suggest that DRG1 has a cancer-promoting effect. However, lower DRG1 expression was found to be associated with advanced stages of cancer and very low survival rates in breast cancer patients [12]. Interestingly, DRG1 knockdown *in vitro* has little or no effect on cell growth, motility, and invasion of breast cancer cells [12]. Low DRG1 expression was also associated with a more aggressive kind of colorectal cancer, while high DRG1 expression enhanced irinotecan resistance in colorectal cancer [13]. Therefore, DRG1 apparently plays different roles in diverse type of cancers.

N6-methyladenosine (m6A)—which occurs on RNA adenine (A) in the consensus sequence, RRACH (R = G or A, H = A, C, or U)—is one of the most prominent modifications in poly-adenylated mRNAs and long-noncoding RNAs. m6A is not randomly distributed in RNA; it is usually present at the 3′ untranslated region (3′UTR), stop codon, and internal exons. m6A methylation is a dynamically reversible process regulated by methyltransferases, demethylases, and function managers, also termed as ‘writers’, ‘erasers’ and ‘readers’, respectively [14,15]. Writers mainly include METTL3, METTL14, and WTAP; they are in charge of RNA methylation. FTO and ALKBH5 are the chief erasers that mediate the demethylation process of m6A. Readers are a group of proteins that recognize m6A-modified RNAs and then regulate multiple processes of RNA, such as RNA stability, translation, splicing, and transport [14,15]. Since m6A modification plays an important role in gene expression, its dysfunction has been associated with various diseases, for instance, cancer [15,16]. Using methylated RNA immunoprecipitation sequencing (MeRIP-seq), a recent study found that m6A was enriched in pluripotency-related genes in doxorubicin-resistant OS cells [17]. In addition, METTL3 has been found to be up-regulated in OS tissues and cells [18]. METTL3 knockdown suppressed the proliferation, migration, and invasion of OS cells. These reports suggest that m6A modification has pathological manifestations in OS.

Our study initially investigated the expression of DRG1 in OS tissues and cell lines and the regulatory effects of DRG1 on various biological processes of OS cells, including viability, apoptosis, colony formation, migration, and invasion. Moreover, we explored the effect of m6A modification on DRG1 expression in OS cells. The results would help us improve our understanding of OS pathogenesis.

## Materials and methods

### Bioinformatics analysis

The present study initially analyzed Drg1 expression in normal bone (*n*=2) and SARC tissues (461) using data from TCGA dataset (http://gepia.cancer-pku.cn/detail.php?gene=Drg1). Since the samples of normal bone tissues were too small, Student’s *t* test failed to generate outcome. Alternatively, the present study analyzed Drg1 expression in osteoblasts and OS tissues using GEO datasets. Osteoblasts are regarded as the normal bone cells. GSE14359 dataset (https://www.ncbi.nlm.nih.gov/geo/query/acc.cgi?acc=GSE14359) provides the data of Drg1 expression in osteoblasts (*n*=2) and OS tissues (*n*=18) among which eight OS tissues were obtained from patients with metastatic OS. Similarly, due to too small samples from osteoblasts, Student’s *t* test was unable to conduct the analysis. GSE33383 dataset (https://www.ncbi.nlm.nih.gov/geo/query/acc.cgi?acc=GSE33383) provides the data of Drg1 expression in osteoblasts (*n*=3) and OS tissues (*n*=84), however no significant difference was found in Drg1 expression between osteoblasts and OS tissues dependent on Student’s *t* test.

To understand Drg1 function in cells, we initially used String web (https://string-db.org/) to find proteins that interacted with DRG1. In this web, the minimum required interaction score was set as the medium confidence (0.400), the maximum number of interactors was less than 100, and the active interaction sources were choice from textmining, experiments, databases, co-expression, neighborhood, gene fusion, and co-occurrence. Moreover, we used Genemania web (http://genemania.org/) in which all genes or proteins that have co-expression, genetic interactions, or physical interactions with DRG1 are included in the present study. All genes and proteins screened out from String and Genemania webs were entered into a GO Enrichment analysis web (https://david.ncifcrf.gov/) with parameters set as official gene symbol, gene list and molecular functions and biological processes.

### Collection of tissue samples

Cancerous (*n*=28) and paracancerous tissues (*n*=28) of OS were obtained from patients undergoing surgery for OS at The First Affiliated Hospital of Guangxi Medical University (Guangxi, China) between 2017 and 2019. Morphologically normal bone tissues that were more than 5 cm from cancerous tissues were used as paracancerous tissues. Patients included in the present study had not received systemic venous chemotherapy, radiotherapy or immunotherapy. All patients were informed about the purpose of using these tissue samples and written informed consent was obtained for the same. The clinicopathological characteristics of patients with OS are shown in Table 1. The study protocol was approved by the Human Ethics Committee of Guilin Hospital and the study was performed in accordance with the Declaration of Helsinki.

**Table 1:** Relationship between *Drg1* expression and the clinicopathological features of OS patients

Clinicopathological features	***n***	High expression	Low expression	*P*-value
Gender				0.356
Male	16	9	7	
Female	12	5	7	
Age (years)				0.574
≤21	21	11	10	
>21	7	3	4	
Tumor size (cm^3^)				0.017
≤20	13	4	9	
>20	15	10	5	
Metastasis				0.069
Yes	14	8	6	
No	14	6	8	
Lung metastasis				0.402
Yes	9	5	4	
No	19	9	10	
Clinical stage				0.033
I/II	12	4	8	
III	16	9	5	

### PCR assay

An RNA-extraction agent, TRIzol (Invitrogen, CA, U.S.A.), was used to extract mRNA from the tissues and cells (to be used in subsequent cell studies). cDNA was synthesized using a commercial kit (Sigma–Aldrich, Co. LLC, St. Louis, MO, U.S.A.), via the reverse transcription of mRNA (1 μg). Real-time PCR was performed using this cDNA in an ABI 7300 Real-Time PCR System (Applied Biosystems, Foster City, CA, U.S.A.). The thermocycling sequence was set as: 95°C for 30 s, followed by 40 cycles of 95°C for 5 s, and 60°C for 30 s. The primer sequences used were DRG1 forward, 5′-TGGAGGTCCAGGAGAAGGTTT-3′ and DRG1 reverse, 5′-GCCACTGCAATGACTTGACG-3′; GAPDH forward, 5′-GGGAAGGTGAAGGTCGGAGT-3′ GAPDH reverse, 5′-TTGAGGTCAATGAAGGGGTCA-3′. DRG1 expression level was normalized to that of GAPDH using the 2^−ΔΔ*C*_T_^ method.

### Western blot assay

Proteins were extracted from tissues and cells using the HEPES lysis buffer (Invitrogen) and then boiled with SDS/PAGE loading buffer (Invitrogen) for protein denaturation. Proteins were separated using SDS/PAGE, followed by transferring on to nitrocellulose filter membrane. Target proteins in the NC membrane were examined via immunoblotting using the following primary antibodies: anti-DRG1 (Abcam, Cambridge, U.K.) anti-Cyclin B1 (Abcam), anti-CDK1 (Abcam), and anti-GAPDH (Abcam) as well as appropriate secondary antibodies. Protein level was quantified using a gel documentation and analysis system. GAPDH was used as the internal control to verify basal expression levels and equal protein loading.

### Cell lines

hFOB1.19 (SV40-immortalized normal osteoblastic cell lines) and OS cell lines (Saos-2, U2OS, MG63, and 143B) were obtained from the Chinese Academy of Sciences Cell Bank (Shanghai, China). hFOB 1.19 cells were cultured in F12/DMEM medium (Gibco, CA, U.S.A.) supplemented with 2.5 mM l-glutamine, and fetal bovine serum to a final concentration of 10%. The OS cells were cultured in DMEM containing 10% FBS (Gibco), 100 units/ml of penicillin–streptomycin (Invitrogen) in a humidified incubator with 5% CO_2_.

### Cell transfection

DRG1 was silenced using three siRNAs targeting DRG1, synthesized by Shanghai GenePharma (China). Their sequences were 5′-GACCAUACGUUGGAGGAUGTT-3′ (siRNA1), 5′-GGUAGAGGUCGUCAAGUCATT-3′ (siRNA2), and 5′-GGCCAGUUACCAGAUUACATT-3′ (siRNA3). The sequences of siRNAs targeting METTL3, ELAVL1, and IGF2BP2 were obtained from previous studies [19–21]. All siRNAs and scramble siRNA (a non-targeting siRNA) were transfected into the cells using the Lipofectamine 2000 kit (Invitrogen), following the manufacturer’s protocol.

### Cell viability assay

Cell Counting Kit-8 (CCK-8, Dojindo, Japan) was used to assess cell viability. As per the manufacturer’s instructions, cells were washed with phosphate-buffered saline (PBS) and then cultured in a culture medium containing 10% CCK-8 solution at 37°C for 3–6 h. Optical density (OD) at 450 nm was detected using a microplate reader (BIOTEK, Vermont, U.S.A.).

### Apoptosis assay

Two fluorescent dyes, Annexin V-FITC (5 μl, BD Biosciences, San Diego, CA, U.S.A.) and propidium iodide (PI, 5 μl, BD Biosciences) were added to the cells. After incubation for 15 min, cells (1 × 10^4^) were examined using a flow cytometer (FACS Calibur, Becton Dickinson, San Jose, CA, U.S.A.) to assess the apoptosis rate.

### Cell cycle assay

Cell cycle progression was determined via PI (BD Biosciences) staining, using a flow cytometer. In brief, cells were fixed with 70% cold ethanol at 4°C overnight. The cells were rehydrated and washed twice with ice-cold PBS and incubated with 10 mg/ml RNase (Fermentas, Shanghai, China) at 37°C. Subsequently the cell cycle was observed via PI staining of the nuclei and analyzed using FACS flow cytometry (Becton Dickinson).

### Cell colony formation assay

Cells were cultured for 2 weeks until colonies were clearly observed. After fixing with methanol for 10 min, colonies were stained with 0.1% Crystal Violet solution (Sigma–Aldrich) for 5 min. The colonies were counted using the ImageJ software.

### Wound-healing assay

Cell migration capacity was evaluated using wound healing assay. A 200 μl sterile pipette tip was used to create a ‘scratch-wound’ on the cell monolayer. Cells were then cultured in a serum-free medium for 48 h. The cell migration rate was calculated based on the movement of cells from the position of initial placement to the final distance, using the equation: (initial distance – final distance)/initial distance ×100.

### Transwell assay

A transwell (8-mm-pore size, Millipore) was used for the evaluation of cell invasion ability. The upper chamber was initially coated with Matrigel (BD, U.S.A.). Serum-free medium and complete medium were added to the upper and lower chambers, respectively. After culturing for 24 h, the bottom of the upper membrane was stained using 0.1% Crystal Violet in 4% paraformaldehyde (PFA). The invading cells were quantified by counting ten random fields under a light microscope (E200; Nikon Corporation, Tokyo, Japan).

### m6A-IP-qPCR

Total RNA was extracted from cells using TRIzol (Invitrogen) and then treated with DNase I to remove DNA contamination. The purified mRNA was fragmented to approximately 100 nucleotides using an RNA fragmentation reagent (Ambion) and then subjected to immunoprecipitation using protein A magnetic beads that were conjugated with an anti-m6A antibody (Abcam). Enrichment of m6A-containing transcript segments was measured using RT-qPCR.

### mRNA stability assay

Actinomycin D was added at a concentration of 5 mg/ml to inhibit intracellular RNA synthesis. At stipulated time points, total RNA from the cells was obtained using TRIzol reagent (Invitrogen). RNA quantities were determined using RT-qPCR.

### Statistical analysis

Statistical analysis was completed using GraphPad Prism 6 software and results were expressed as mean ± standard deviation. Comparisons between experimental group and control group were performed using Student’s *t* test or one-way ANOVA. A *P*-value of <0.05 was considered as statistically significant.

## Results

### DRG1 was up-regulated in OS tissues and cell lines

**Figure 1:**
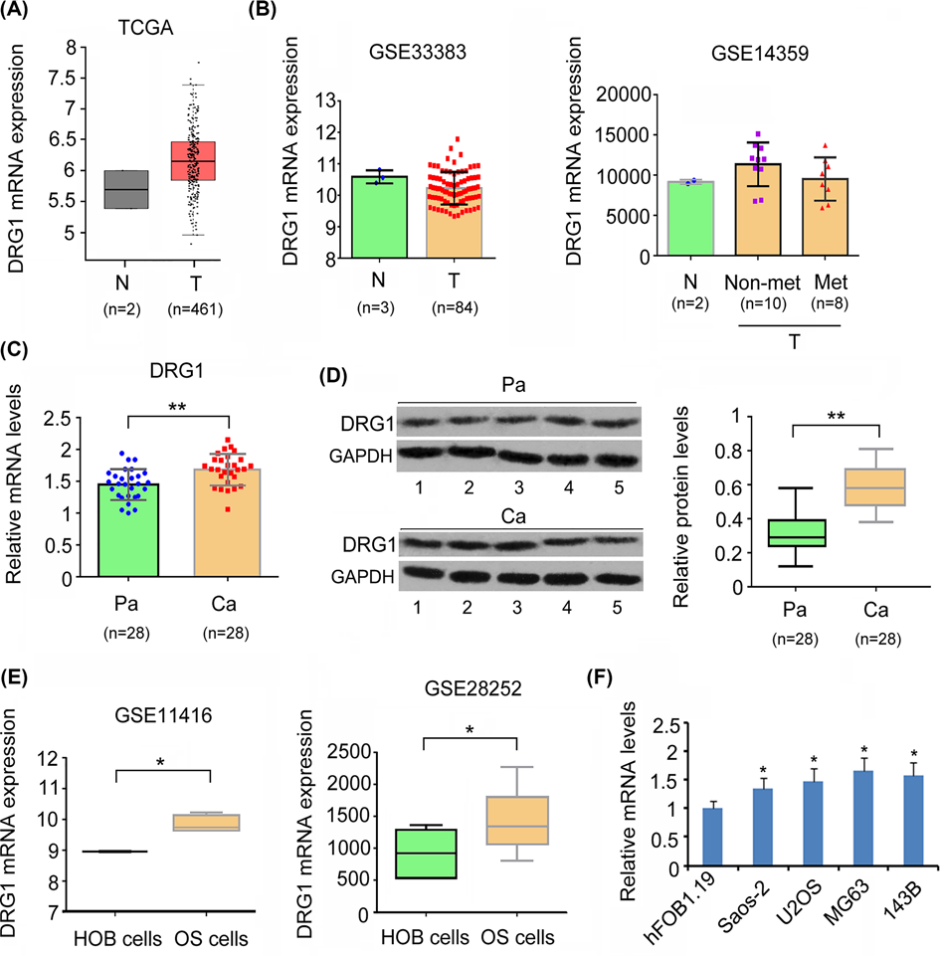
DRG1 was up-regulated in OS tissues and cell lines Data from TCGA (**A**) and GEO GSE33383 and GSE14359 datasets (**B**) showed no significant difference between OS and normal bone tissues in DRG1 expression. N: normal bone tissues or osteoblasts; T: OS tissues; Non-met: OS without metastasis; Met: OS with metastasis. OS cancer tissues showed higher expression of DRG1 mRNA (**C**) and protein (**D**) than paracancerous tissues. Representative bands of Western blot from five OS and paracancerous tissues were shown in (D). Pa: paracancerous tissues; Ca: OS cancer tissues. **P<0.01 vs. paracancerous tissues. GEO datasets showed that increased expression of DRG1 in OS cell lines compared with osteoblasts (**E**). *P<0.05 vs. osteoblasts. DRG1 was highly expressed in Saos-2, U2OS, MG63, and 143B cells compared with hFOB1.19 cells (**F**). *P<0.05 vs. hFOB1.19 cells.

Bioinformatics analysis was performed to evaluate DRG1 expression in OS tissues and osteoblasts. TCGA (Figure 1A) and GSE14359 (Figure 1B) datasets just provide limited samples of normal bone tissues, therefore Student’s *t* test failed to generate outcome. Data from GSE33383 dataset showed no significant difference between OS tissues and osteoblasts in DRG1 expression (Figure 1B). Because the samples of normal osteoblasts were too small in both TCGA and GEO datasets, the present study used paracancerous tissues as normal tissues. Results from both, PCR and Western blot assay revealed higher expression of DRG1 in OS cancerous tissues than in paracancerous tissues (*P*<0.01, Figure 1C,D). GEO datasets showed increased expression of DRG1 in OS cell lines compared with that in osteoblasts (*P*<0.05, Figure 1C). This result was observed in our study also. We found that DRG1 was highly expressed in Saos-2, U2OS, MG63 and 143B cells compared with hFOB1.19 cells. MG63 and 143B cells were used in further studies, since they showed higher expression of DRG1 than the other OS cell lines.

To explore the association of DRG1 to clinicopathologic characters of OS, we first allocated the patients with OS to DRG1-high (*n*=14) and -low groups (*n*=14), depending on whether DRG1 expression level was higher or lower than the average level of DRG1 mRNA in all the patients. High expression level of DRG1 was associated with big tumor size (*P*<0.05) and advanced clinical stages of OS (*P*<0.05, Table 1).

### The regulatory effects of DRG1 on various hallmarks of OS cells

**Figure 2:**
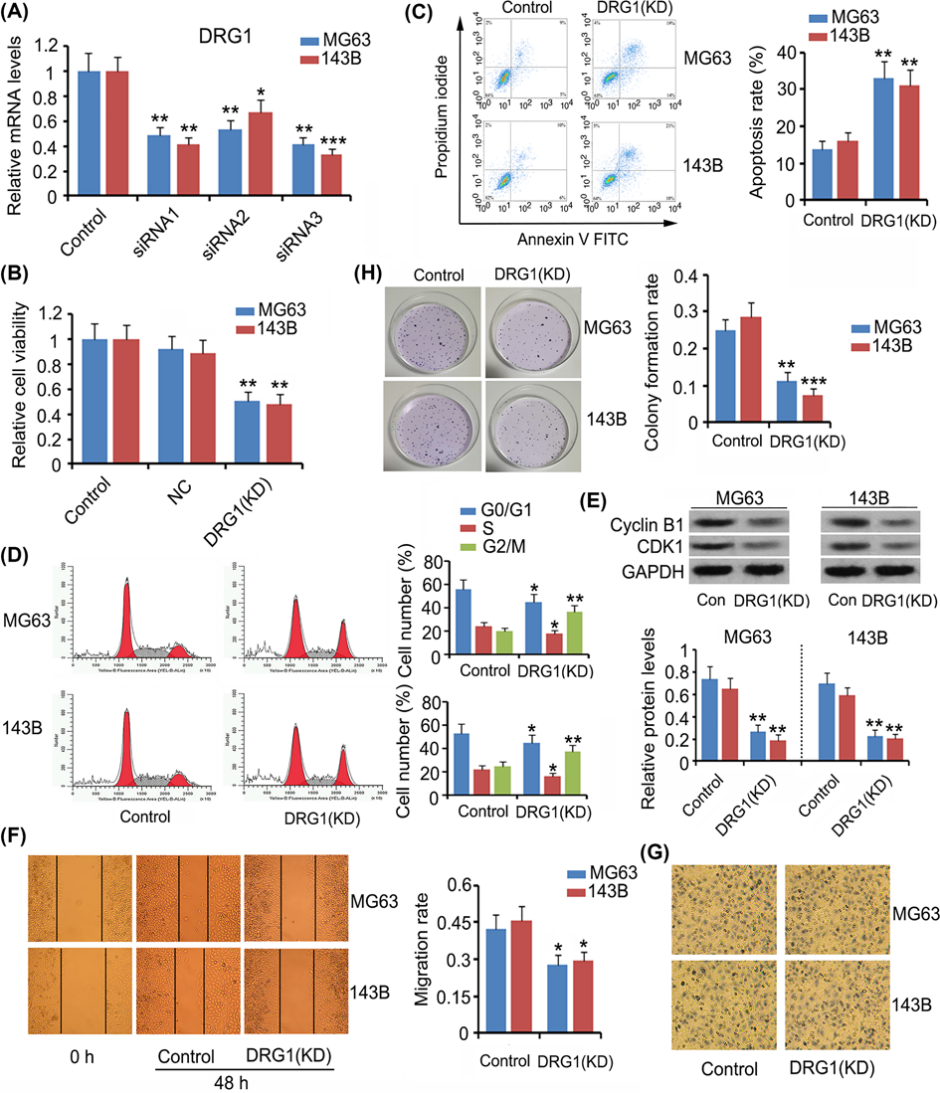
The regulatory effects of DRG1 on various hallmarks of OS cells To knock down DRG1, the present study designed three siRNAs to target DRG1. siRNA3 showed the highest efficiency in DRG1 knockdown, thus it was used in further study (**A**). After DRG1 knockdown, cell viability (**B**), apoptosis rate (**C**), cell cycle distribution (**D**), expression of Cycline B and CDK1 (**E**), cell migration (**F**), invasion (**G**), and colony formation rate (**H**) were evaluated. Silencing of Drg1 resulted in decreased viability and inhibition of the migration and colony formation abilities of OS cells; it also resulted in cell cycle arrest in the G_2_/M stage and induced apoptosis. *P<0.05, **P<0.01, and ***P<0.001 vs. control.

We chose to not overexpress DRG1 in OS cells, because DRG1 was already highly expressed in the cells, and the gene sequence of DRG1 was very long, thus making it difficult to be inserted into an expression vector. Alternatively, we knocked down DRG1 to determine its influence on the hallmarks of OS cells. We designed three siRNAs to target DRG1. All of them significantly reduced DRG1 expression in MG63 and 143B cells (*P*<0.05, *P*<0.01, or *P*<0.001, Figure 2A). siRNA3 showed the highest efficiency in DRG1 knockdown, thus it was used in further studies. Silencing DRG1 significantly inhibited MG63 and 143B cell viability (*P<*0.01, Figure 2B), while increasing apoptosis rate (*P<*0.01, Figure 2C). Data from flow cytometry showed that the proportion of MG63 and 143B cells in G_2_/M phase increased after DRG1 knockdown (*P<*0.01, Figure 2D) and in turn, the proportions of cells in G_0_/G_1_ and S phases decreased (*P<*0.05). DRG1 deficiency was correlated to lower cell migration (*P<*0.05, Figure 2E) and colony formation rates (*P*<0.01 or *P<*0.001, Figure 2G,H) in comparison with those in the control group, but cell invasion ability was not affected by DRG1 reduction (Figure 2F).

**Figure 3:**
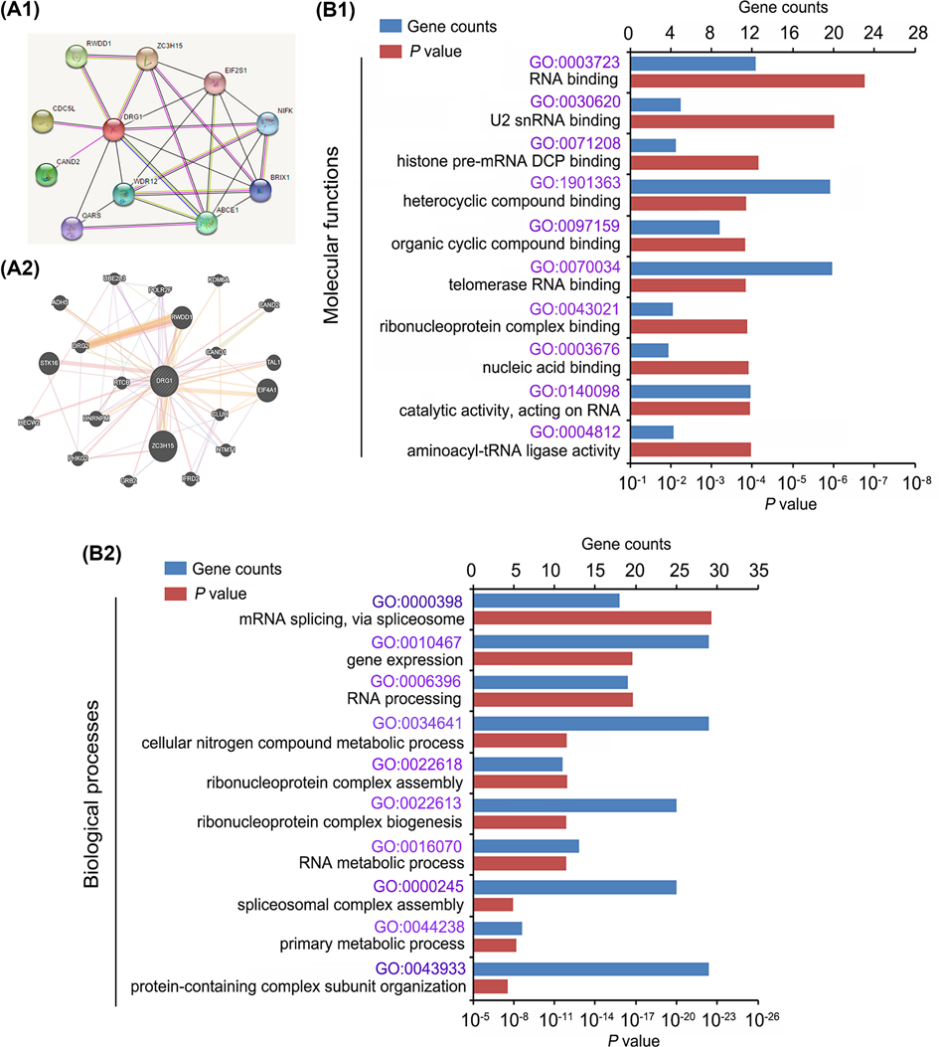
Bioinformatics analysis revealed DRG1 biological functions Using data from https://string-db.org/ (**A1**) and http://genemania.org/ (**A2**), bioinformatics analysis revealed proteins that interact with DRG1 and genes whose expression was closely associated with DRG1 expression. The figures (A1,2) only showed the part of the genes and proteins. GO Enrichment analysis of these proteins and genes (https://david.ncifcrf.gov/) showed the potential molecular functions of DRG1 (**B1**) and primary biological processes affected by DRG1 (**B2**).

Using data from https://string-db.org/ (Figure 3A1) and http://genemania.org/ (Figure 3A2), we performed bioinformatics analysis to find proteins that interacted with DRG1 and genes whose expression was closely associated with DRG1 expression. These proteins and genes were postulated to be linked to the biological functions of DRG1. Therefore, we performed GO Enrichment analysis of these proteins and genes (https://david.ncifcrf.gov/). The potential molecular functions of DRG1 were theorized to include RNA binding (such as U2 snRNA binding and telomerase RNA binding), nucleic acid binding, histone pre-mRNA DCP binding, and so on (Figure 3B1). The primary biological processes affected by DRG1 were RNA processing, such as mRNA splicing and RNA metabolic process, cellular nitrogen compound metabolic process and ribonucleoprotein complex assembly and biogenesis (Figure 3B2).

### DRG1 expression was affected by METTL3 and ELAVL1 in an m6A-dependent manner in OS cells

**Figure 4:**
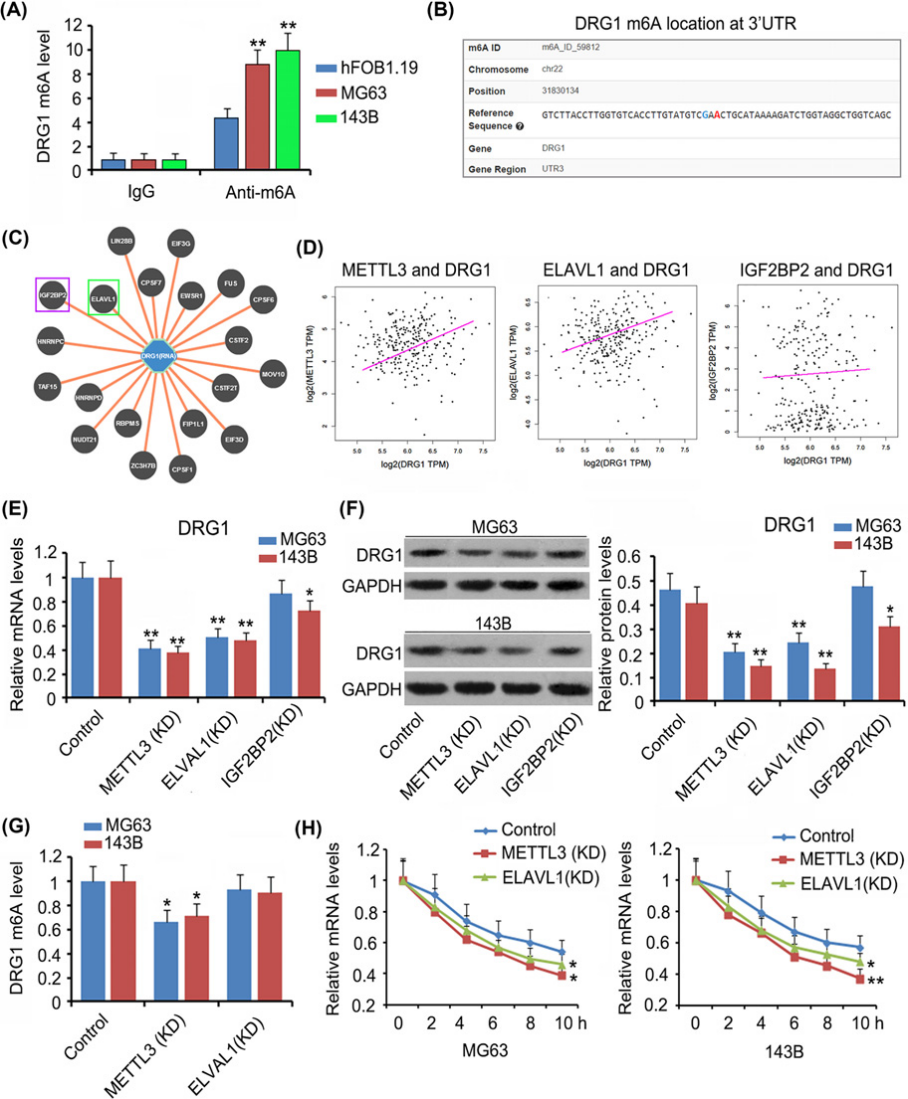
DRG1 expression was affected by METTL3 and ELAVL1 in m6A-dependent manner in OS cells As indicated by M6A-IP-qPCR assay, m6A level of DRG1 mRNA was higher in MG63 and 143B cells than in hFOB1.19 cells (**A**). **P<0.01 vs. hFOB1.19 cells. An m6A prediction website (http://m6avar.renlab.org/) showed an m6A position at 3′UTR of DRG1 mRNA (**B**) and proteins that showed high affinity to DRG1 mRNA (**C**). Among the proteins, ELAVL1 and IGF2BP2 are m6A readers. Bioinformatics analysis (http://gepia.cancer-pku.cn/) further showed that DRG1 expression was positively correlated to METTL3 and ELAVL1 expression, but not to IGF2BP2 expression (**D**). Silencing METTL3 and ELAVL1 significantly decreased both mRNA (**E**) and protein levels of DRG1 (**F**) in MG63 and 143B cells. Depletion of IGF2BP2 only decreased DRG1 mRNA and protein levels in 143B cells. m6A level of DRG1 mRNA was undermined after METTL3 knockdown (**G**). *P<0.05 and **P<0.01 vs. control. The stability of DRG1 mRNA was impaired after both METTL3 and ELAVL1 knockdown (**H**). *P<0.05 and **P<0.01 vs. 0 h. Abbreviation: KD, knockdown.

As indicated by m6A-IP-qPCR assay, m6A levels of DRG1 mRNA were higher in MG63 and 143B cells than in hFOB1.19 cells (*P*<0.01 or *P*<0.001, Figure 4A). An m6A prediction website (http://m6avar.renlab.org/) showed the m6A position at the 3′UTR of DRG1 mRNA (Figure 4B) and proteins with high affinity to DRG1 mRNA (Figure 4C). Among these proteins, ELAVL1 and IGF2BP2 are m6A readers. Bioinformatics analysis (http://gepia.cancer-pku.cn/) further revealed that DRG1 expression was positively correlated to METTL3 (r = 0.0235, *P<*0.01) and ELAVL1 (r = 0.0212, *P<*0.05) expression, but not to IGF2BP2 expression (Figure 4D). We knocked down METTL3, ELAVL1, and IGF2BP2 to further determine their regulatory effects on DRG1 expression. Silencing METTL3 and ELAVL1 significantly decreased both mRNA and protein levels of DRG1 (*P<*0.01, Figure 4E,F) in MG63 and 143B cells. Depletion of IGF2BP2 decreased DRG1 mRNA and protein levels only in 143B cells. m6A level of DRG1 mRNA was undermined after METTL3 knockdown (*P<*0.05, Figure 4G). The stability of DRG1 mRNA was hampered after both METTL3 and ELAVL1 knockdown (*P<*0.05 or *P*<0.01 at 10 h, Figure 4H).

## Discussion

The present study found that DRG1 was abnormally up-regulated in OS tissue compared with paracancerous tissues, and that up-regulated DRG1 was associated with big tumor size and advanced clinical stages of OS. Previous studies performed microarray and high throughput sequencing assays to ascertain differentially expressed genes between OS and osteoblasts. The data were documented in TCGA and GEO databases. However, no significant difference was observed in DRG1, which is probably due to very small number of osteoblast samples. The number of osteoblast samples in TCGA dataset was less than three. Results from GSE11416 and GSE28252 datasets and our study showed that DRG1 was significantly up-regulated in OS cell lines when compared with normal human osteoblasts. Aberrantly expressed DRG1 has been observed in other types of cancers [10–13], but up-regulated DRG1 is not always associated with the worst types of cancer. For example, high *Drg1* expression is more likely associated with a less aggressive and indolent colorectal cancer [13]. These results suggest that DRG1 has varying regulatory effects in different cancers.

The present study demonstrated that silencing DRG1 inhibited cell viability, arrested cell cycle and triggered cell apoptosis. These data suggest that DRG1 exerted cancer-promoting effects in OS. Liu et al. reported the interaction of DRG1 with kinetochore proteins (e.g. CENPN) and spindle assembly checkpoint proteins, such as Mad2 and BubR1 in lung adenocarcinomas [10]. Since these proteins are required for cell cycle and chromosomal segregation, it is possible that DRG1 also participates in the regulation of these functions. In our study, DRG1 knockdown increased the percentage of OS cells in G_2_/M phases. The normal cell cycle progresses sequentially through G_0_/G_1_, S, G_2_ and M phases for cell proliferation. However, cell cycle is highly regulated by cell cycle checkpoints. Activation of such checkpoints triggers a transient or sustained cell cycle arrest, therefore blocking the cell cycle progression is considered as an effective strategy to hinder proliferation of cancer cells. Cell cycle arrest resulted in an increase in the percentage of cells at one or more phases of the cell cycle. Since G_2_ and M phases are difficult to be segregated using flow cytometric analysis via cell PI staining, it was necessary to detect the levels of cyclin B1 and CDK1 which are G_2_/M cell cycle regulatory proteins and help cells pass through the checkpoint between G_2_ and M phases. This study demonstrated that DRG1 knockdown down-regulated cyclin B1 and CDK1 and increased apoptosis. These data collectively suggest that DRG1 knockdown caused the arrest in G_2_/M phase, and this arrest probably resulted in apoptosis. A study found that knockdown of ribosome biogenesis regulatory protein homolog (RRS1) also trapped colorectal cancer cells in the G_2_/M phase and finally resulted in their apoptosis [22].

DRG1 knockdown was associated with the suppression of migration and colony formation of OS cells, whereas it had no effect on cell invasion. Cell migration assay was used to evaluate cell mobility that is required for cancer metastasis. Cell invasion assay assesses not only cell mobility, but also levels of matrix metalloproteins (MMPs) that are secreted by cancer cells. MMPs are responsible for the degradation of extracellular matrix. A loose connection between the surrounding cells aids cancer invasion and metastasis [23]. In cell invasion assay, the upper chamber was initially coated with Matrigel, therefore the cancer cells needed to secret MMPs to dissolve Matrigel before passing through the membrane of the upper chamber. Our data suggested that DRG1 promotes mobility of OS cells, while it does not influence secretion of MMPs by OS cells. A higher colony formation rate suggests stronger adaptability of cancer cells to new environment after metastasis. Although DRG1 knockdown undermined colony formation and migration rate of OS cells, DRG1 expression was not associated with its metastasis in the clinical analysis. Given that the clinical analysis was performed on a relatively small number of OS samples, further study is recommended to evaluate the association between DRG1 expression and OS metastasis using more number of clinical samples.

Previous studies have reported interactions of DRG1 with many oncogenic transcription factors such as c-myc, SCL, and TAL1 [4,24,25]. This study performed PPI (protein–protein interaction) analysis to find the proteins that interacted with DRG1, along with gene enrichment analysis to explore the biological functions influenced by these proteins. The results suggest that DRG1 is involved in various processes that regulate gene expression. Therefore, DRG1 knockdown probably affected expression of a large number of genes. This hypothesis needs to be further verified using microarray or high throughput sequencing assay in future studies.

The present study confirmed that m6A modification mediated by METTL3 and ELAVL1 promoted the stability of DRG1 mRNA, thereby increasing DRG1 protein level. Increased expression of METTL3 has been observed in OS tissues, implying that m6A level is higher in OS tissues as compared with that in the corresponding normal tissues. As indicated by M6A-IP-qPCR, traditional PCR and Western blot assays, silencing METTL3 decreased m6A, mRNA and protein levels of DRG1. These data suggest that METTL3 promotes DRG1 expression in an m6A-dependent manner. Previous study found that METTL3 overexpression stimulated m6A modification of hepatoma-derived growth factor (HDGF) mRNA. m6A reader IGF2BP3 further enhanced HDGF mRNA stability, resulting in increased HDGF expression. HDGF contributed to the proliferation and metastasis of gastric cancer [26]. In addition, YAP and MALAT1 expression was also increased by METTL3 through m6A modification [27]. They promoted the growth, metastasis, and drug resistance of non-small-cell lung cancer [27]. METTL3 being an m6A writer, the regulatory effects of METTL3 on biological processes of mRNA rely on m6A readers. The present study found two m6A readers, ELAVL1 and IGF2BP2, bound to m6A sequence of DRG1, but DRG1 expression is positively correlated to that of ELAVL1, but not to IGF2BP2. Moreover, ELAVL1 knockdown significantly decreased DRG1 expression in both MG63 and 143B cells, while IGF2BP2 knockdown only undermined DRG1 expression in 143B cells. Therefore, ELAVL1 might be a more important reader for the m6A sequence of DRG1. After ELAVL1 knockdown, the stability of DRG1 mRNA decreased with reduction in DRG1 mRNA level. Various studies have confirmed the role of ELAVL1 in mRNA stability in m6A-dependant manner [28,29].

In summary, high expression level of DRG1 was associated with big tumor size and advanced clinical stages of OS. Silencing *Drg1* inhibited the viability, migration and colony formation of OS cells, arrested cell cycle in G2/M stage and induced apoptosis. Knockdown of METTL3 was associated with decreased m6A and mRNA levels of *Drg1*. ELAVL1 knockdown impaired the mRNA stability of DRG1, thereby decreasing both, mRNA and protein levels of DRG1. In conclusion, DRG1 exerted cancer-promoting effects on OS, and up-regulated DRG1 in OS was induced by METTL3 and ELAVL1 in an m6A-dependent manner.

## Abbreviations

CCK-8: cell counting kit-8
DRG: developmentally regulated GTP-binding protein
ELAVL1: ELAV Like RNA Binding Protein 1
HDGF: hepatoma-derived growth factor
IGF2BP2: insulin like growth factor 2 MRNA binding protein 2
METTL3: methyltransferase like 3
MMP: matrix metalloprotein
m6A: N6-methyladenosine
OS: osteosarcoma
PBS: phosphate-buffered saline
PI: propidium iodide
TCGA: The Cancer Genome Atlas
3′UTR: 3′ untranslated region
